# Social Vulnerability and Compliance With World Health Organization Advice on Protective Behaviors Against COVID-19 in African and Asia Pacific Countries: Factor Analysis to Develop a Social Vulnerability Index

**DOI:** 10.2196/54383

**Published:** 2024-08-13

**Authors:** Suladda Pongutta, Viroj Tangcharoensathien, Kathy Leung, Heidi J Larson, Leesa Lin

**Affiliations:** 1 International Health Policy Program Ministry of Public Health Muang Nonthaburi Thailand; 2 WHO Collaborating Centre for Infectious Disease Epidemiology and Control School of Public Health, Li Ka Shing Faculty of Medicine The University of Hong Kong Hong Kong SAR China; 3 Laboratory of Data Discovery for Health Limited (D24H) Hong Kong Science Park Hong Kong SAR China; 4 The University of Hong Kong Shenzhen Hospital Shenzhen China; 5 Department of Infectious Disease Epidemiology London School of Hygiene and Tropical Medicine London United Kingdom; 6 Centre for the Evaluation of Vaccination Vaccine & Infectious Disease Institute University of Antwerp Antwerp Belgium; 7 Department of Health Metrics Sciences University of Washington Seattle, WA United States

**Keywords:** social vulnerability Index, COVID-19, protective behavior, Africa, Western Pacific, social vulnerability, African countries, Western countries, predict, predicts, prediction, propensity, baseline data, Factor analysis, polychoric, varimax rotation, protective, behaviour, behaviours, preventive, precautionary, linear regression, predictability, Omicron, sociodemographic, media use, communication, health risk perception, socioeconomic

## Abstract

**Background:**

COVID-19 protective behaviors are key interventions advised by the World Health Organization (WHO) to prevent COVID-19 transmission. However, achieving compliance with this advice is often challenging, particularly among socially vulnerable groups.

**Objective:**

We developed a social vulnerability index (SVI) to predict individuals’ propensity to adhere to the WHO advice on protective behaviors against COVID-19 and identify changes in social vulnerability as Omicron evolved in African countries between January 2022 and August 2022 and Asia Pacific countries between August 2021 and June 2022.

**Methods:**

In African countries, baseline data were collected from 14 countries (n=15,375) during the first Omicron wave, and follow-up data were collected from 7 countries (n=7179) after the wave. In Asia Pacific countries, baseline data were collected from 14 countries (n=12,866) before the first Omicron wave, and follow-up data were collected from 9 countries (n=8737) after the wave. Countries’ socioeconomic and health profiles were retrieved from relevant databases. To construct the SVI for each of the 4 data sets, variables associated with COVID-19 protective behaviors were included in a factor analysis using polychoric correlation with varimax rotation. Influential factors were adjusted for cardinality, summed, and min-max normalized from 0 to 1 (most to least vulnerable). Scores for compliance with the WHO advice were calculated using individuals’ self-reported protective behaviors against COVID-19. Multiple linear regression analyses were used to assess the associations between the SVI and scores for compliance to WHO advice to validate the index.

**Results:**

In Africa, factors contributing to social vulnerability included literacy and media use, trust in health care workers and government, and country income and infrastructure. In Asia Pacific, social vulnerability was determined by literacy, country income and infrastructure, and population density. The index was associated with compliance with the WHO advice in both time points in African countries but only during the follow-up period in Asia Pacific countries. At baseline, the index values in African countries ranged from 0.00 to 0.31 in 13 countries, with 1 country having an index value of 1.00. The index values in Asia Pacific countries ranged from 0.00 to 0.23 in 12 countries, with 2 countries having index values of 0.79 and 1.00. During the follow-up phase, the index values decreased in 6 of 7 African countries and the 2 most vulnerable Asia Pacific countries. The index values of the least vulnerable countries remained unchanged in both regions.

**Conclusions:**

In both regions, significant inequalities in social vulnerability to compliance with WHO advice were observed at baseline, and the gaps became larger after the first Omicron wave. Understanding the dimensions that influence social vulnerability to protective behaviors against COVID-19 may underpin targeted interventions to enhance compliance with WHO recommendations and mitigate the impact of future pandemics among vulnerable groups.

## Introduction

SARS-CoV-2 infections can still pose a significant threat, especially for individuals with underlying health conditions and older adults [1], and 45% of those recovering from COVID-19, regardless of severity, experience sequelae (eg, fatigue, breathlessness, impaired sleep, reduction in grey matter brain thickness, other impairments to daily activities) [2,3]. Although immunity against COVID-19 has developed in a significant proportion of the global population and the impact of the disease has diminished, it is unlikely to be completely eradicated in the near future [4,5]. Furthermore, resurgences are possible [6] due to waning immunity against COVID-19 and the evolution of new variants [7,8].

The World Health Organization (WHO) recommended personal protective behaviors to prevent COVID-19 infection, including receiving the full COVID-19 vaccine course, social distancing, maintaining proper indoor ventilation, wearing a mask if at risk, regular handwashing, covering coughs and sneezes, and staying home when feeling unwell [9,10]. These protective behaviors played an important role in minimizing COVID-19 transmission, morbidity, and mortality [11,12], with failure to follow the WHO advice contributing to the spread of SARS-CoV-2 [13]. However, adherence to these social measures has often been challenging for many due to various factors, including vaccine hesitancy, economic disruption, and unemployment [13-16].

The capabilities (which include the abilities, qualities, and willingness) of individuals to comply with the WHO advice necessitate a collaborative effort between governments and individuals. Governments must ensure universal access to the necessary infrastructure and health services needed for COVID-19 control, including ensuring access to vaccines, masks, and hand sanitizers and adapting public spaces to facilitate safe distancing between individuals [17]. Additionally, these measures should be accompanied by effective public communication strategies to promote vaccine uptake and other personal protective behaviors and to address misinformation [17]. Individuals, on the other hand, are required to accept COVID-19 vaccines and to adapt their lifestyles or health behaviors to protect themselves from serious illnesses due to COVID-19 and limit its spread [9]. Inequitable capabilities to comply with this advice vary between countries and across individuals in a country [18-20]. For example, low- and middle-income countries may face challenges accessing vaccines and other health facilities [19,21], while maintaining social distancing and access to clean water are not often possible for many low-income groups [18,20,22]. Individual beliefs and perceptions regarding COVID-19 also vary [23,24].

Evidence shows that vulnerable populations were disproportionately affected by the pandemic [25], so identifying vulnerable populations who have limited ability to protect themselves from infection is crucial to ensure equitable health outcomes [26]. Individuals’ positions within their physical and social environments can determine their ability to cope with hazards or harm, reflecting their level of social vulnerability [27]. A social vulnerability index (SVI) is a composite index that integrates the contributions of diverse social determinants that determine people’s vulnerability to a certain threat, and it is widely used as a tool to identify vulnerable populations susceptible to and most affected by specific threats [28]. An SVI can be advantageous, as it simplifies complex data, facilitates cross-country and temporal comparisons, and permits weighting of the most important dimensions [29].

COVID-19 community vulnerability indices have been developed in some countries, for example, in Africa [30], the United States [31], Kenya [32], and the United Kingdom [33]. These indices are defined by community- and country-level metrics, rather than individual-level metrics. However, these indices are unlikely to reflect individuals’ capabilities to comply with the WHO advice because they do not include variables that influence behaviors, such as individuals’ socioeconomic status, perceptions and attitudes toward COVID-19, and trust in governments [24,34-36]. Furthermore, the variables used for these indices are often constant or change only slightly over time, making these indices unlikely to capture the dynamic behavioral responses of individuals during the different waves of COVID-19, which is essential information for developing an effective response.

Of all COVID-19 variants, Omicron has been responsible for the largest number of confirmed COVID-19 cases worldwide [37]. Globally, the WHO Western Pacific region reported the highest number of confirmed cases from Omicron, while the WHO Africa region had the lowest [37,38]. In the WHO Africa region, Omicron was identified as the fourth wave of COVID-19 in mid-November 2021. The number of confirmed cases reached its peak between December 2021 and January 2022, before declining to baseline in April 2022 [37,39]. In the WHO Western Pacific region, Omicron cases exhibited fluctuating patterns throughout 2022, with the first wave occurring between January and April, the second wave occurring between July and September, and the third wave occurring in December [37,38]. Understanding the patterns of and changes in social vulnerability that predict an individual’s capability to comply with the WHO advice in these countries will provide beneficial information to guide COVID-19 prevention, especially among vulnerable populations.

In this study, we developed an SVI to predict individuals’ propensity to adhere to the WHO advice on protective behaviors against COVID-19 and to identify changes in social vulnerability as Omicron evolved in African countries between January 2022 and August 2022 and Asia Pacific countries between August 2021 and June 2022.

## Methods

### Conceptual Framework of Social Vulnerability to Compliance With WHO Advice on COVID-19 Protective Behaviors

An SVI should encompass the influences of multiple key factors of a threat or hazard to identify vulnerable groups who are most likely to be affected by the threat or hazard [40]. Current literature indicates that COVID-19 protective behaviors are associated with sociodemographic status (eg, sex, age, income, education, occupation), media use and communication, personal health risk perception, trust in the government, trust in health personnel, socioeconomic status (eg, gross domestic product [GDP] per capita), and access to public health infrastructure (eg, information system, cold chain in vaccine transport, medical facilities per capita, health care access and quality) [21,24,34,35,41-44]. Characteristics of both households and populations, such as access to handwashing facilities and urban population density, have also been used as indicators for an SVI for COVID-19 morbidity and mortality [32].

**Figure 1:**
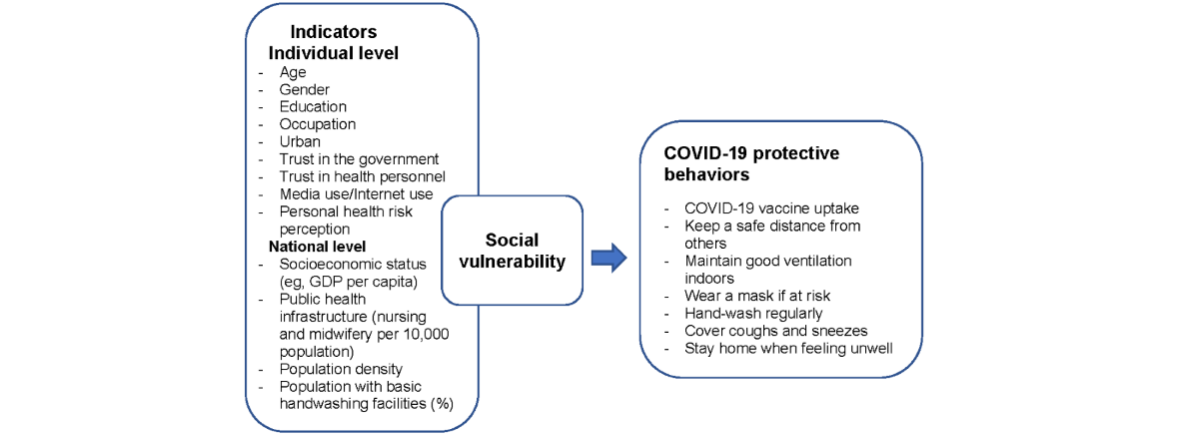
Conceptual framework to construct a social vulnerability index that reflects compliance with World Health Organization advice on COVID-19 protective behaviors. GDP: gross domestic product.

In our SVI construction models, we included factors associated with COVID-19 protective behaviors, morbidity, and mortality. Figure 1 illustrates the framework used to guide the analysis of this study.

### Variables and Data Sources 

#### Variables and Data Sources for SVI Construction

Individual-level variables used to analyze the SVI for African countries were sourced from 2 nationally representative surveys conducted by the Vaccine Confidence Project in January 2022 (baseline, during the first wave of Omicron) and August 2022 (follow-up, after the first wave of Omicron). Baseline data were collected from 14 countries (n=15,375): Cameroon, Cote d'Ivoire, Democratic Republic of the Congo, Ghana, Kenya, Liberia, Mali, Niger, Nigeria, Senegal, Sierra Leone, South Africa, South Sudan, and Uganda. Follow-up data, weighted by age and gender, were collected from 7 countries (n=7179): Cameroon, Democratic Republic of the Congo, Kenya, Nigeria, Senegal, South Africa, and Uganda. These variables encompassed age, gender, education, employment status, urbanicity, trust in the government, trust in health personnel, COVID-19 risk perception, and media use and communication. National-level variables used in this analysis included GDP per capita in 2021 for both waves [45], population density in 2022 for both waves [46], the percentage of the population with basic handwashing facilities at home (latest updated) [47], nursing and midwifery personnel per 10,000 population (latest updated) [48], and health-adjusted life expectancy (HALE) at birth (latest updated) [49].

Variables at the individual level available for the analysis of the SVI for Asia Pacific countries were obtained from 2 nationally representative surveys carried out by the Vaccine Confidence Project from June 2021 to August 2021 (baseline, before the first wave of Omicron) and May 2022 to June 2022 (follow-up, after the first wave of Omicron). Baseline data were collected from 14 countries (n=12,866): China, Cambodia, Vietnam, Lao People's Democratic Republic, Japan, South Korea, Malaysia, Philippines, Mongolia, Fiji, Papua New Guinea, Solomon Islands, Tonga, and Vanuatu. Follow-up data (weighted by age and gender) were collected from 9 countries (n=8737): Cambodia, Vietnam, Lao People's Democratic Republic, Japan, South Korea, Malaysia, Philippines, Mongolia, and Papua New Guinea. Variables used in the SVI construction included age, gender, education, employment status, and COVID-19 risk perception. Variables at the national level used in this analysis were GDP per capita in 2020 for baseline and in 2021 for follow-up [45], population density in 2021 for baseline and in 2022 for follow-up [46], nursing and midwifery personnel per 10,000 population (latest updated) [48], and HALE at birth (latest updated) [49]. Variables included in the SVI construction and sources of data are presented in Table S1 in Multimedia Appendix 1.

#### Variables and Data Sources for Computation of the Score for Compliance With WHO Advice on COVID-19 Protective Behavior

We computed scores, which reflect individuals’ capabilities to comply with the WHO advice, using the COVID-19 protective behavior data from the Vaccine Confidence Project’s surveys carried out in the African and Asia Pacific countries listed in the previous section. We identified relative changes in the scores between baseline and follow-up. Variables used in the score computation included doses of COVID-19 vaccines, keeping a safe distance from others, maintaining proper indoor ventilation, wearing a mask if at risk, regular handwashing, covering coughs and sneezes, and staying home when feeling unwell. Each variable was assigned a score of 0 to 3, from least improved (0, indicating negative change or no improvement) to most improved (3, indicating greatest positive change or most improvement). Scores for all variables were summed to obtain the score for compliance with WHO recommendations on COVID-19 protective behavior for each individual. Table 1 illustrates the measurements for COVID-19 protective behaviors and computation of the scores. Since the variables at baseline and during follow-up in Asia Pacific countries were slightly different, the scores were normalized to enable the comparison between the 2 waves.

**Table 1:** Variables used to compute compliance with World Health Organization advice on COVID-19 protective behavior score in African countries (AFCs) and Asia Pacific countries (APCs).

Variable	Score range	Codes	AFCs		APCs	
			Baseline^a^	Follow-up^a^	Baseline^a^	Follow- up^b^
1. Willingness to accept COVID-19 vaccines and have had COVID-19	0 to 3	0=Definitely don’t accept COVID-19 vaccines/lean toward don’t; 1=leaning toward accepting COVID-19 vaccines; 2=Definitely accept; 3=Have had COVID-19 vaccine	No	No	Yes	No
2. Number of doses of COVID-19 vaccines received	0 to 3	0=0 dose; 1=1 dose; 2=2 doses; 3=>2 doses	Yes	Yes	No	Yes
3. Wearing a face mask	0 to 3	0=Not at all/less regularly than before the COVID-19 pandemic; 1=About the same; 2=A little more regularly; 3=A lot more regularly	Yes	Yes	Yes	Yes (merged questions 3 and 4)
4. Covering your mouth and nose when sneezing/coughing	0 to 3	0 = Not at all/less regularly than before the COVID-19 pandemic; 1=About the same; 2=A little more regularly; 3=A lot more regularly	Yes	Yes	Yes	Yes (merged questions 3 and 4)
5. Washing my hands	0 to 3	0=Not at all/less regularly than before the COVID-19 pandemic; 1=About the same; 2=A little more regularly; 3=A lot more regularly	Yes	Yes	Yes	Yes
6. Having guests in your house	0 to 3	3=Not at all; 2=Less regularly than before the COVID-19 pandemic; 1=About the same; 0=A little more regularly/a lot more regularly	Yes	Yes	Yes	Yes (merged questions 6 and 7)
7. Gathering socially in large groups	0 to 3	3=Not at all; 2=Less regularly than before the COVID-19 pandemic; 1=About the same; 0=A little more regularly/a lot more regularly	Yes	Yes	Yes	Yes (merged questions 6 and 7)
8. Staying home when feeling unwell	0 to 3	0=Not at all/less regularly than before the COVID-19 pandemic; 1=About the same; 2=A little more regularly; 3=A lot more regularly	No	No	No	Yes
9. Keeping physical distance from others	0 to 3	0=Not at all/less regularly than before the COVID-19 pandemic; 1=About the same; 2=A little more regularly; 3=A lot more regularly	Yes	Yes	Yes	No
10. Ensuring that spaces I share with others are well ventilated	0 to 3	0=Not at all/less regularly than before the COVID-19 pandemic; 1=About the same; 2=A little more regularly; 3=A lot more regularly	No	No	No	Yes

^a^Total score range: 0-21.

^b^Total score range: 0-18.

### Management of Missing Data

The percentage of missing data for most variables at the individual level ranged from 0.2% to 3.7%. Single imputation was used to address missing values for education, employment status, urbanicity, trust in the government, trust in health personnel, COVID-19 risk perception, media use and communication, and health behaviors, whereby missing values were replaced with the most frequent value for that variable.

### Analysis

Data analysis was conducted according to the practical guide for social vulnerability assessment by the United Nations Development Programme [28] and the variable reduction approach for SVI construction [29]. Variables potentially influencing COVID-19 protective behaviors were included in separate factor analysis models using polychoric correlation with varimax rotation approach with a loading cutoff of 0.3 [50] to identify influential factors and specific variable domains (see results in Figure S1 in Multimedia Appendix 1). Influential factors with an eigenvalue of more than 1 were selected, adjusted for cardinality, summed (equal weights), and min-max normalized to develop a composite index of individual social vulnerability for each region at baseline and follow-up. A *t* test was used to test the difference between mean SVI at baseline and mean SVI during follow-up in each country.

For each wave of data collection, multiple linear regression analysis was used to assess the association between the index as the independent variable and compliance with WHO advice on COVID-19 protective behavior score as the dependent variable, which was adjusted for sociodemographic characteristics that were excluded from the construction of the SVI and country clustering effects. The analyses were conducted using STATA 17. The SVIs of compliance with the WHO advice were spatially presented using Excel365 (Microsoft Corp).

### Ethical Considerations

The surveys were approved by the Institutional Review Board at the London School of Hygiene and Tropical Medicine (LSHTM 26636) and the Human Research Ethics Committee of the University of Hong Kong (EA230420). Participants were informed of voluntary participation, the privacy and confidentiality protection policy, and the right to withdraw from the survey at any time. Written consent was obtained from all participants, and the use of the data for this study was allowed. This study was performed according to the principles of the Declaration of Helsinki.

## Results

### Characteristics of Individuals and Countries Included in the SVI Construction

Table 2 presents the characteristics of the indicators included in the factor analysis models for constructing the SVI. Almost all the characteristics of the African and Asia Pacific countries were different, except for the proportion of men to women and the proportion of participants who did not believe in the threat of COVID-19; however, the proportion of participants with a high concern of being infected by COVID-19 was slightly lower in African countries. In African countries, there was a higher proportion of younger individuals and a higher proportion of participants with master’s or higher degrees but a lower proportion of participants with bachelor’s degrees, a lower proportion of participants working full time, lower GDP per capita, fewer nurses and midwives per 10,000 population, and lower population density compared with Asia Pacific countries. In African countries, the proportion of participants having high trust in the government and health care workers decreased, and the information sources individuals had high access to (everyday use >40%) were television, radio, and word of mouth in the local community.

**Table 2:** Characteristics of individuals and countries included in the social vulnerability index construction.

Indicators				African countries				Asia Pacific countries		
				Baseline		Follow-up		Baseline		Follow-up
Countries, n				14		7		14		9
Participants, n				15,375		7179		12,866		8737
**Age (years), n (%)**										
	18-24			4251 (27.6)		2092 (29.1)		2405 (18.7)		1632 (18.7)
	25-34			4923 (32)		2368 (33)		3476 (27)		2158 (24.7)
	35-44			2980 (19.4)		1384 (19.3)		2835 (22)		1793 (20.5)
	45-54			1656 (10.8)		758 (10.6)		2081 (16.2)		1407 (16.1)
	≥55			1565 (10.2)		577 (8)		2069 (16.1)		1747 (20)
Gender (male), n (%)				7672 (49.9)		3597 (50.1)		6471 (50.3)		4427 (50.7)
**Education, n (%)**										
	No formal education			3039 (19.8)		584 (8.1)		588 (4.5)		329 (3.8)
	Primary education			2934 (19.1)		1207 (16.8)		1403 (10.9)		898 (10.3)
	Secondary education			5678 (36.9)		3056 (42.6)		5392 (41.4)		3525 (40.4)
	Vocational postsecondary education and other			567 (3.7)		195 (2.7)		1076 (8.4)		715 (8.2)
	Bachelor			988 (6.4)		691 (9.6)		4066 (31.6)		2905 (33.3)
	Master and PhD			2167 (14.1)		1446 (20.1)		412 (3.2)		357 (4.1)
**Employment status, n (%)**										
	Unemployed (no income)			2998 (19.5)		1545 (21.5)		1303 (10.1)		841 (9.6)
	Retired, student, stay-at-home parent (no own income from work)			4261 (27.7)		1812 (25.3)		3123 (24.3)		2093 (24)
	Working part time, self-employed, jobs other than working full time (irregular income)			2971 (19.3)		1487 (20.7)		1647 (12.8)		1663 (19)
	Working full time (regular income)			5145 (33.5)		2335 (32.5)		6793 (52.8)		4140 (47.4)
Area of residence (urban), n (%)				7597 (49.4)		3930 (54.7)		N/A^a^		N/A
**Trust in the government, n (%)**										
	Not at all			3032 (19.7)		1679 (23.4)		N/A		N/A
	Not much			2064 (13.4)		1087 (15.2)		N/A		N/A
	Somewhat			2859 (18.6)		1890 (26.3)		N/A		N/A
	A lot			7420 (48.3)		2523 (35.1)		N/A		N/A
**Trust in health care personnel (%)**										
	Not at all			1017 (6.6)		484 (6.7)		N/A		N/A
	Not much			1303 (8.47)		637 (8.9)		N/A		N/A
	Somewhat			3103 (20.2)		2078 (29)		N/A		N/A
	A lot			9952 (64.7)		3980 (55.4)		N/A		N/A
**COVID-19 risk perception, n (%)**										
	**A. What level of threat does COVID-19 pose to you?**									
		No threat	3063 (19.9)		1141 (15.9)		N/A		N/A	
		Very low threat	1026 (6.7)		470 (6.5)		N/A		N/A	
		Fairly low threat	680 (4.4)		442 (6.2)		N/A		N/A	
		Moderate threat	1070 (6.9)		786 (11)		N/A		N/A	
		Fairly high threat	1401 (9.1)		890 (12.4)		N/A		N/A	
		Very high threat	8135 (52.9)		3450 (48)		N/A		N/A	
	**B. How concerned are you about getting COVID-19?**									
		Not at all	2692 (17.5)		1234 (17.2)		N/A		N/A	
		A little	2652 (17.2)		1484 (20.7)		N/A		N/A	
		Moderate	1920 (12.5)		1087 (15.1)		N/A		N/A	
		Very	8111 (52.8)		3374 (47)		N/A		N/A	
	**C. How strongly do you agree or disagree that the threat from COVID-19 is exaggerated?**									
		Strongly disagree	N/A		N/A		2411 (18.7)		1256 (14.4)	
		Disagree	N/A		N/A		4971 (38.6)		2763 (31.6)	
		Agree	N/A		N/A		3612 (28.1)		3804 (43.5)	
		Strongly agree	N/A		N/A		1872 (14.6)		914 (10.5)	
**Media use and communication, n (%)**										
	**Television**									
		Never	3639 (23.7)		983 (13.7)		N/A		N/A	
		Less than once a month	813 (5.3)		323 (4.5)		N/A		N/A	
		At least once a month	957 (6.2)		443 (6.2)		N/A		N/A	
		At least once a week	2522 (16.4)		1154 (16.1)		N/A		N/A	
		Every day	7444 (48.4)		4276 (59.6)		N/A		N/A	
	**Radio**									
		Never	2580 (16.8)		1115 (15.5)		N/A		N/A	
		Less than once a month	917 (6)		464 (6.5)		N/A		N/A	
		At least once a month	1107 (7.2)		578 (8.1)		N/A		N/A	
		At least once a week	3056 (19.9)		1393 (19.4)		N/A		N/A	
		Every day	7715 (50.2)		3629 (50.5)		N/A		N/A	
	**Printed media**									
		Never	8894 (57.8)		3300 (45.9)		N/A		N/A	
		Less than once a month	1405 (9.1)		793 (11)		N/A		N/A	
		At least once a month	1414 (9.2)		877 (12.2)		N/A		N/A	
		At least once a week	1943 (12.6)		1173 (16.3)		N/A		N/A	
		Every day	1719 (11.2)		1036 (14.4)		N/A		N/A	
	**Word of mouth in the local community**									
		Never	2042 (13.3)		630 (8.8)		N/A		N/A	
		Less than once a month	801 (5.2)		361 (5)		N/A		N/A	
		At least once a month	1106 (7.2)		579 (8.1)		N/A		N/A	
		At least once a week	2903 (18.9)		1352 (18.8)		N/A		N/A	
		Every day	8523 (55.4)		4257 (59.3)		N/A		N/A	
	**Social media**									
		Never	6268 (40.8)		2080 (29)		N/A		N/A	
		Less than once a month	702 (4.6)		299 (4.2)		N/A		N/A	
		At least once a month	695 (4.5)		392 (5.5)		N/A		N/A	
		At least once a week	1953 (12.7)		1056 (14.7)		N/A		N/A	
		Every day	5757 (37.4)		3352 (46.7)		N/A		N/A	
	**Internet**									
		Never	6954 (45.2)		2234 (31.1)		N/A		N/A	
		Less than once a month	744 (4.8)		381 (5.3)		N/A		N/A	
		At least once a month	835 (5.4)		518 (7.2)		N/A		N/A	
		At least once a week	1997 (13)		1116 (15.6)		N/A		N/A	
		Every day	4845 (31.5)		2930 (40.8)		N/A		N/A	
Population using the internet (%), range				Not included		Not included		11-98		11-98
GDP^b^ per capita (US $), range				486.95-5741.64		577.20-7055		1577.91-39,918.17		1625.20-39,312.70
Nursing and midwifery personnel per 10,000 population, range				2.23-49.74		3.63-49.74		4.53-119.5		4.53-119.5
HALE^c^ at birth (years), range				52.9-59.4		54.1-59.4		57.1-74.1		57.1-74.1
Population with basic handwashing facilities at home (%), range				3.0-44.0		19.2-44.4		N/A		N/A
Population density (per km^2^), range				17.0-234.3		43.7-239.9		2.2-531.5		2.2-531.3

^a^N/A: not applicable.

^b^GDP: gross domestic product.

^c^HALE: health-adjusted life expectancy.

### Social Vulnerability Index (SVI)

#### SVI in African Countries

The factor analysis of baseline data for African countries identified 3 influential factors that explained 88.9% of the total variance shared among the included variables. Factor 1 was the main contributor of total variance (43.4%), followed by factor 2 (29.5%) and factor 3 (16%). Factor 1 was defined by indicators related to literacy and media use (ie, age, education, urbanicity, and media use [TV, print media, social media, internet]). Factor 2 encompassed indicators relevant to trust including trust in the government and trust in health care workers. Factor 3 included indicators related to country income and infrastructure, which consisted of GDP per capita, population with basic handwashing facilities at home, HALE, and nursing and midwifery personnel per 10,000 population. Age had a negative correlation with factor 1, while all other indicators had positive correlations.

**Figure 2:**
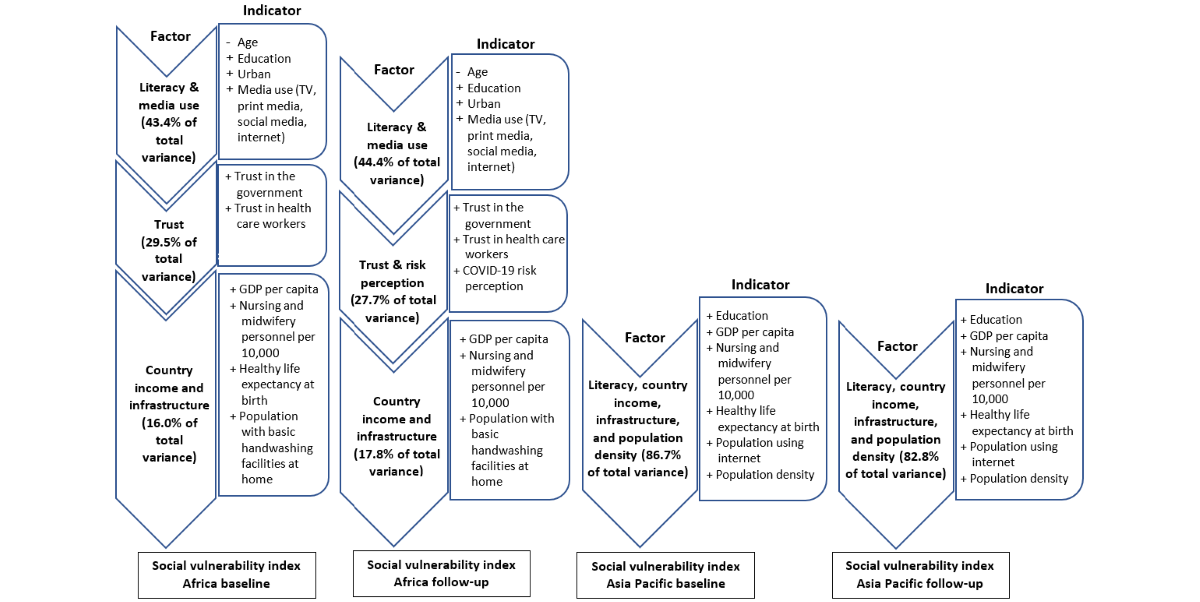
Factors and correlated indicators identified by factor analyses at baseline and follow-up in Africa and Asia Pacific. GDP: gross domestic product; +: positive loading; -: negative loading.

Follow-up data from African countries identified 3 factors that explained 89.8% of total variance. Similar to the baseline, factor 1 contributed most to total variance (44.4%), followed by factor 2 (27.7%) and factor 3 (17.8%). Overall, these factors included indicators similar to those identified from baseline data, except that COVID-19 risk perception was additionally included in factor 2 and HALE was excluded from factor 3. Age was the only indicator with a negative correlation with factor 1 (see Figure 2). Factor loadings are presented in Figure S1 in Multimedia Appendix 1.

**Figure 3:**
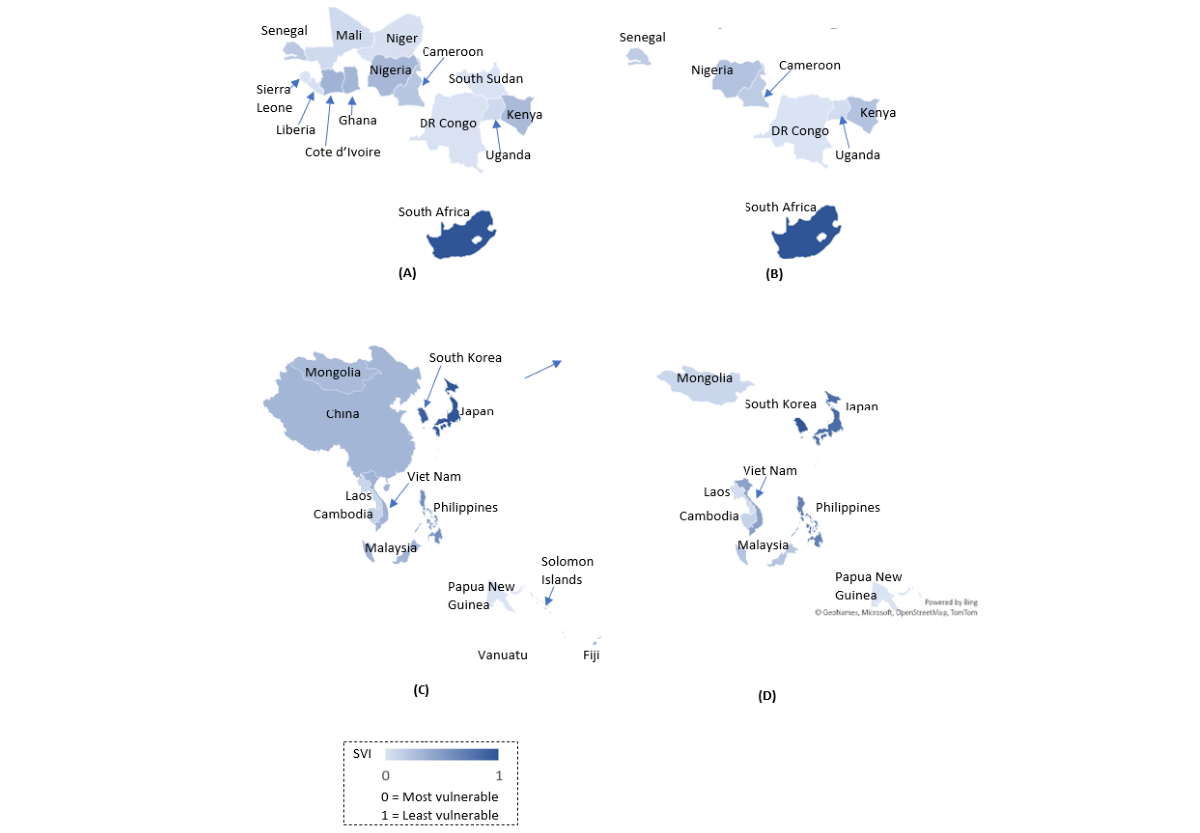
Spatial presentation of the social vulnerability index (SVI) on compliance with World Health Organization advice on COVID-19 protective behaviors and patterns of changes in SVIs among (A) African countries at baseline (during Omicron), (B) African countries at follow-up (after Omicron), (C) Asia Pacific countries at baseline (during Omicron), and (D) Asia Pacific countries at follow-up (after Omicron). DR: Democratic Republic.

The mean SVIs in African countries at baseline ranged from 0.00 (extremely vulnerable) to 1.00 (least vulnerable). Most African countries (13 of 14 countries) were extremely vulnerable, with mean SVIs ranging from 0.00 to 0.31. When comparing the periods during and after Omicron’s peak, mean SVIs further decreased in 6 of 7 extremely vulnerable countries, while mean SVIs in the least vulnerable country remained the same (see Table 3 and Figure 3).

**Table 3:** Social vulnerability index (SVI) of compliance with World Health Organization advice on COVID-19 protective behaviors and changing patterns of the SVI in African and Asia Pacific countries at baseline and follow-up.

Country			Baseline				Follow-up					Change, %			*P* value^a^
			Survey respondents, n		SVI^b^, mean (SD)		Survey respondents, n		SVI^b^, mean (SD)						
**Africa**															
	Cameroon	1058		0.18 (0)		1008		0.19 (0)		–5.56			<.001		
	Cote d’Ivoire	1129		0.31 (0)		N/A^c^		N/A		N/A			N/A		
	Democratic Republic of the Congo	1190		0.01 (0)		1021		0 (0)		–100			<.001		
	Ghana	1094		0.29 (0)		N/A		N/A		N/A			N/A		
	Kenya	1030		0.24 (0)		1078		0.27 (0)		–4.17			<.001		
	Liberia	1011		0.03 (0)		N/A		N/A		N/A			N/A		
	Mali	1152		0.06 (0)		N/A		N/A		N/A			N/A		
	Niger	1032		0.02 (0)		N/A		N/A		N/A			N/A		
	Nigeria	1024		0.24 (0)		1008		0.29 (0)		–4.17			<.001		
	Senegal	1139		0.18 (0)		1001		0.18 (0)		–11.11			<.001		
	Sierra Leone	1104		0 (0)		N/A		N/A		N/A			N/A		
	South Africa	1171		1.00 (0)		982		1.00 (0)		0			.38		
	South Sudan	1219		0.09 (0)		N/A		N/A		N/A			N/A		
	Uganda	1022		0.06 (0)		1081		0.06 (0)		–16.67			<.001		
All African countries			15,375		0.20 (0.25)		7179		0.28 (0.30)		N/A			<.001	
**Asia Pacific**															
	China	2000		0.23 (0)		N/A		N/A		N/A			N/A		
	Cambodia	1000		0 (0)		1000		0 (0)		–34.22			<.001		
	Vietnam	1044		0.05 (0)		1003		0.06 (0)		7.23			<.001		
	Lao People's Democratic Republic	1000		0.03 (0)		1000		0.02 (0)		–8.76			<.001		
	Japan	1040		1.00 (0)		1066		1.00 (0)		0			.001		
	South Korea	1133		0.79 (0)		1155		0.89 (0)		12.62			<.001		
	Malaysia	1000		0.22 (0)		1000		0.25 (0)		12.34			<.001		
	Philippines	1000		0.05 (0)		1000		0.05 (0)		9.87			<.001		
	Mongolia	1000		0.06 (0)		1000		0.08 (0)		20.25			<.001		
	Fiji	521		0.09 (0)		N/A		N/A		N/A			N/A		
	Papua New Guinea	552		0.02 (0)		513		0.03 (0)		25.07			<.001		
	Solomon Islands	527		0.02 (0)		N/A		N/A		N/A			N/A		
	Tonga	517		0.08 (0)		N/A		N/A		N/A			N/A		
	Vanuatu	532		0.03 (0)		N/A		N/A		N/A			N/A		
All Western Pacific countries			12,866		0.23 (0.31)		8737		0.29 (0.38)		N/A			<.001	

^a^Significance set at *P*<.05.

^b^0 represents the most vulnerable, and 1 represents the least vulnerable in the region.

^c^N/A: not applicable.

#### SVI in APCs

At baseline, 1 factor accounting for 86.7% of the total variance was identified. This factor was defined by 6 indicators, including education, GDP per capita, HALE, nursing and midwifery personnel per 10,000 population, population using the internet, and population density. All indicators had positive correlations with the factor.

During the follow-up, our factor analysis identified 1 factor that explained 82.8% of the total variance. This factor was defined by the same set of indicators with the same direction of relationships as at baseline.

Figure 2 illustrates the factors and indicators used for constructing the SVI of compliance with WHO advice on COVID-19 protective behaviors. Factor loadings are shown in Figure S1 in Multimedia Appendix 1.

In Asia Pacific countries, the pattern of mean SVIs at baseline was similar to that in African countries in terms of range. Of 14 countries, 12 countries had mean SVIs ≤0.23, while only 2 countries had mean SVIs of 0.79 (South Korea) and 1.00 (Japan). The majority of Asia Pacific countries experienced positive changes in their SVI at follow-up, while the 2 most vulnerable countries (Cambodia and Lao People's Democratic Republic) experienced negative changes after Omicron’s peak in the region in 2022 (see Table 3 and Figure 3).

### Compliance With WHO Advice on COVID-19 Protective Health Behaviors

In African countries, when comparing the scores for compliance with COVID-19 protective health behaviors at baseline and follow-up, the score decreased in 5 countries (Cameroon, Democratic Republic of the Congo, Nigeria, South Africa, and Uganda). The score decreased most in Democratic Republic of the Congo, followed by Nigeria, Uganda, Cameroon, and South Africa. Positive changes were found in Kenya and Senegal.

In Asia Pacific countries, 3 countries had reduced compliance scores at the follow-up survey, with the greatest decrease in Mongolia, followed by Cambodia and Papua New Guinea. Positive changes in compliance scores were observed in Vietnam, Lao People's Democratic Republic, Japan, South Korea, Malaysia, and the Philippines (see Table 4).

**Table 4:** Scores for compliance with World Health Organization advice on COVID-19 protective behaviors and changing patterns in the compliance scores in African and Asia Pacific countries at baseline and follow-up.

Country			Baseline					Follow-up					Change, %
			Survey respondents, n		Compliance score, mean (SD)^a^		Survey respondents, n			Compliance score, mean (SD)^a^			
**Africa**													
	Cameroon	1058		8.77 (4.04)		1008			8.56 (3.78)		–2.39		
	Cote d’Ivoire	1129		10.71 (3.88)		N/A^b^			N/A		N/A		
	Democratic Republic of the Congo	1190		10.08 (3.57)		1021			5.74 (4.01)		–43.06		
	Ghana	1094		8.71 (4.01)		N/A			N/A		N/A		
	Kenya	1030		9.66 (3.46)		1078			11.22 (4.30)		16.15		
	Liberia	1011		9.87 (3.71)		N/A			N/A		N/A		
	Mali	1152		6.45 (3.87)		N/A			N/A		N/A		
	Niger	1032		13.36 (3.40)		N/A			N/A		N/A		
	Nigeria	1024		12.69 (3.97)		1008			8.48 (3.25)		–33.18		
	Senegal	1139		6.18 (3.99)		1001			7.75 (4.48)		25.40		
	Sierra Leone	1104		7.37 (4.08)		N/A			N/A		N/A		
	South Africa	1171		11.65 (4.44)		982			11.62 (3.65)		–0.26		
	South Sudan	1219		6.35 (2.92)		N/A			N/A		N/A		
	Uganda	1022		11.83 (4.09)		1081			10.94 (4.03)		–7.52		
All African countries			15,375		9.47 (4.46)		7179			9.21 (4.43)		N/A	
**Western Pacific**													
	China	2000		0.77 (0.10)		N/A			N/A		N/A		
	Cambodia	1000		0.70 (0.17)		1000			0.64 (0.19)		–8.67		
	Vietnam	1044		0.81 (0.10)		1003			0.86 (0.12)		6.10		
	Lao People's Democratic Republic	1000		0.58 (0.21)		1000			0.60 (0.19)		4.10		
	Japan	1040		0.68 (0.22)		1066			0.76 (0.19)		11.48		
	South Korea	1133		0.68 (0.27)		1155			0.75 (0.19)		11.15		
	Malaysia	1000		0.70 (0.24)		1000			0.72 (0.21)		2.56		
	Philippines	1000		0.64 (0.17)		1000			0.67 (0.16)		4.52		
	Mongolia	1000		0.77 (0.13)		1000			0.69 (0.15)		–10.39		
	Fiji	521		0.79 (0.20)		N/A			N/A		N/A		
	Papua New Guinea	552		0.46 (0.19)		513			0.43 (0.18)		–6.49		
	Solomon Islands	527		0.43 (0.16)		N/A			N/A		N/A		
	Tonga	517		0.53 (0.11)		N/A			N/A		N/A		
	Vanuatu	532		0.46 (0.16)		N/A			N/A		N/A		
All Western Pacific countries			12,866		0.67 (0.21)		8737			0.71 (0.19)		N/A	

^a^Mean score for African countries and mean normalized score for Asia Pacific countries.

^b^N/A: not applicable.

### Associations Between SVI and Compliance Scores

Table 5 displays the factors that predicted compliance with WHO advice on COVID-19 protective behaviors in African countries at baseline and follow-up. SVIs in African countries strongly predicted compliance during both waves. At baseline, the mean score for compliance with COVID-19 protective behaviors statistically significantly increased by 2.68 with every unit of SVI and by 0.9 among participants with a high concern about COVID-19 infection. During the follow-up, the mean compliance score increased by 3.64 with every unit of SVI and by 0.49 among men compared with women.

**Table 5:** Associations between the social vulnerability index (SVI) and compliance with World Health Organization advice on COVID-19 protective behaviors among participants from African countries at baseline and follow-up.

Indicator				Coefficient (95% CI)		*P* value^a^
**Baseline (N=15,375)^b^**						
	SVI		2.68 (0.20 to 5.15)		.04	
	**Gender (reference: female)**					
		Male	0.21 (–0.09 to 0.51)		.16	
	**Employment status (reference: unemployed)**					
		Retired/student/stay-at-home parent (no own income)	–0.23 (–1.00 to 0.50)		.51	
		Working part time	–0.76 (–1.60 to 0.09)		.08	
		Working full time	0.24 (–0.44 to 0.92)		.46	
	**COVID-19 risk perception (threat; reference: no threat)**					
		Very low threat	–0.32 (–0.98 to 0.35)		.32	
		Fairly low threat	0.19 (–0.82 to 1.21)		.69	
		Moderate threat	0.04 (–0.84 to 0.92)		.93	
		Fairly high threat	0.21 (–0.75 to 1.18)		.64	
		Very high threat	0.61 (–0.37 to 1.60)		.20	
	**COVID-19 risk perception (concern about being infected; reference: no concern)**					
		Low	0.13 (–0.53 to 0.79)		.67	
		Moderate	0.32 (–0.34 to 0.99)		.31	
		High	0.90 (0.39 to 1.41)		.002	
**Follow-up (N=7179)^c^**						
	SVI		3.64 (1.10 to 6.18)		.01	
	**Gender (reference: female)**					
		Male	0.49 (0.06 to 0.92)		.03	
	HALE^d^		0.46 (–0.40 to 1.32)		.24	
	**Employment status (reference: unemployed)**					
		Retired/student/stay-at-home parent (No own income)	0.46 (–0.08 to 1.01)		.08	
		Working part time	–0.51 (–1.21 to 0.19)		.13	
		Working full time	–0.12 (–1.20 to 0.95)		.79	

^a^Significance set at *P*<.05.

^b^Cameroon, Cote d’Ivoire, Democratic Republic of the Congo, Ghana, Kenya, Liberia, Mali, Niger, Nigeria, Senegal, Sierra Leone, South Africa, South Sudan, and Uganda: R^2^=0.0463, *F*_(13;15,361)_=57.38, *P*<.001.

^c^Cameroon, Democratic Republic of the Congo, Kenya, Nigeria, Senegal, South Africa, and Uganda: R^2^= 0.1137, *F*_(6,7172)_=169.84, *P*<.001.

^d^HALE: health-adjusted life expectancy.

Table 6 shows the predictors of compliance with WHO advice on COVID-19 protective behaviors in Asia Pacific countries at baseline and follow-up. SVIs in Asia Pacific countries predicted compliance scores only during follow-up, when the mean score increased by 1.71 with every unit of SVI. At baseline, the mean scores for compliance among participants aged 35 years to 44 years was higher than scores among those aged 18 years to 24 years (reference group), by 0.54. The mean score among men was lower than that of women, by 0.47. There were negative relationships between the scores and the belief that the threat from COVID-19 is exaggerated.

**Table 6:** Associations between the social vulnerability index (SVI) and compliance with World Health Organization advice among participants from Asia Pacific countries at baseline and follow-up.

Indicator				Coefficient (95% CI)		*P* value^a^
**Baseline (N=12,866)^b^**						
	SVI			0.97 (–1.22 to 3.16)		.36
	**Age (years; reference: 18-24 years)**					
		25-34	0.45 (–0.06 to 0.95)		.08	
		35-44	0.54 (0.02 to 1.06)		.04	
		45-54	0.70 (–0.11 to 1.51)		.08	
		≥55	1.30 (–0.01 to 2.62)		.052	
	**Gender (reference: female)**					
		Male	–0.47 (–0.84 to –0.10)		.02	
	**Employment status (reference: unemployed)**					
		Retired/student/stay-at-home parent (no own income)	0.51 (–0.12 to 1.14)		.11	
		Working part time	0.33 (–0.88 to 1.55)		.57	
		Working full time	1.54 (0.20 to 2.87)		.03	
	**Agree that the threat from COVID-19 is exaggerated (reference: strongly agree)**					
		Agree	–0.68 (–1.24 to –0.13)		.02	
		Disagree	–2.15 (–3.16 to –1.14)		.001	
		Strongly disagree	–1.89 (–3.05 to –0.72)		.004	
**Follow-up (N=8737)^c^**						
	SVI			1.71 (0.05 to 3.37)		.04
	**Age (years; reference: 18-24 years)**					
		25-34	–0.02 (–0.36 to 0.32)		.90	
		35-44	0.19 (–0.30 to 0.69)		.40	
		45-54	0.28 (–0.28 to 0.85)		.28	
		≥55	0.76 (–0.07 to 1.59)		.07	
	**Gender (reference: female)**					
		Male	–0.39 (–0.65 to –0.14)		.008	
	**Employment status (reference: unemployed)**					
		Retired/student/stay-at-home parent (no own income)	0.76 (–0.25 to 1.77)		.12	
		Working part time	0.92 (–0.74 to 2.58)		.24	
		Working full time	1.14 (–0.40 to 2.69)		.13	
	**Area of residence (reference: urban)**					
		Rural	–0.10 (–0.91 to 0.71)		.78	
	**Agree that the threat from COVID-19 is exaggerated (reference: strongly agree)**					
		Agree	–0.06 (–0.75 to 0.63)		.84	
		Disagree	0.61 (–0.43 to 1.65)		.22	
		Strongly disagree	0.52 (–0.31 to 1.36)		.19	

^a^Significance set at *P*<.05.

^b^China, Cambodia, Vietnam, Lao People's Democratic Republic, Japan, South Korea, Malaysia, Philippines, Mongolia, Fiji, Papua New Guinea, Solomon Islands, Tonga, and Vanuatu: R^2^=0.0800, *F*_(12,13)_=18.21, *P*<.001.

^c^Cambodia, Vietnam, Lao People's Democratic Republic, Japan, South Korea, Malaysia, Philippines, Mongolia, and Papua New Guinea: R^2^=0.0687, *F*_(14,8722)_=48.71, *P*<.001.

## Discussion

### Principal Findings

The SVIs that we developed indicated that most countries in Africa and Asia Pacific were extremely socially vulnerable, with significant gaps in SVIs across countries. After the Omicron wave, these social vulnerability gaps became wider due to the deteriorating situations among the most vulnerable populations in both regions. Our results also suggest that compliance with the WHO advice was determined by a combination of multilevel socioeconomic and sociopsychological factors. SVIs in African countries were a strong predictor of scores for compliance with WHO advice both at baseline and during follow-up, while SVIs in Asia Pacific countries showed predictability only at the follow-up survey.

Our findings on social vulnerability, COVID-19, and inequity gaps align with those of previous studies. Individuals with income security and high-level occupations were more likely to adopt COVID-19 protective behaviors than those with lower status [51]. Financially vulnerable populations often face barriers such as limited access to adequate housing conditions, sanitation, personal protective equipment, education, information, job security, quality health care services, and COVID-19 vaccines [21,52,53]. Many studies have indicated that vulnerable groups and low-income countries have fewer capabilities to protect themselves from COVID-19 and recover from negative economic impacts, all of which widens the existing inequity gaps and increases their vulnerability to future public health emergency events [53-57]. This evidence highlights the vicious cycle of social vulnerability and COVID-19.

In African countries, social vulnerability was primarily explained by 3 factors including literacy and media use, trust, and country-level income and infrastructure. Among these factors, literacy and media use had the most significant impact, followed by trust and country-level income and infrastructure. In the aftermath of the Omicron wave, changes in these factors led to a reduction in vulnerability. To ensure equitable recovery and bolster future pandemic preparedness, vital actions encompass launching health literacy campaigns, advancing media literacy, nurturing transparent community engagement to foster trust, investing in health care infrastructure, and creating safety nets for socioeconomic stability.

In Asia Pacific countries, the SVI was intricately linked to critical socioeconomic factors including education, internet accessibility, GDP per capita, health care capacity, healthy life expectancy, and population density. Variations in SVI predominantly stemmed from fluctuations in education, GDP per capita, and population density. To effectively address these dynamics during pandemic recovery, policy priorities should include targeted educational programs to enhance literacy and digital skills, ensure affordable internet access, embrace sustainable urban planning strategies, promote equitable health care access, and introduce programs aiming to reduce income inequality.

The predictability of our SVIs depended on the comprehensiveness of the variables used and the phases of the COVID-19 outbreak. The stronger predictability of the SVIs in African countries can be attributed to the inclusion of a more comprehensive list of relevant variables in its construction. Findings from Asia Pacific countries demonstrate a variation in the predictability of SVIs at different phases of the COVID-19 outbreak. A relationship between Asia Pacific countries’ SVIs and WHO advice compliance scores was found only after Omicron, even though the influential indicators remained consistent both before and after Omicron.

There were changes in parameters that could contribute to the variation in predictability of SVIs in Asia Pacific countries. Upward trends of the SVI and scores for compliance to the WHO advice were found in most Asia Pacific countries, except among certain of the most vulnerable countries. Other studies also reported that COVID-19 protective behaviors increased with number of the COVID-19 cases [58] and social class [59]. Therefore, it is possible that experiencing Omicron triggered better compliance with the WHO advice; however, participants with low levels of social barriers improved their protective behaviors at a higher level than others who had higher social barriers. This resulted in significant differences between the 2 groups.

Our findings improve the understanding of how socioeconomic and various factors determine SVI, how SVI determines compliance with WHO recommendations, and how the SVI and compliance scores varied temporally as the pandemic evolves. This could lead to better policy response in identifying vulnerable populations, minimizing their social barriers, mobilizing external assistance, and monitoring progress of implementation. Regular assessment of SVIs across population groups contributes to specific and effective interventions.

### Limitations

This study also has limitations. First, certain important variables for the construction of the SVI were absent, especially in Asia Pacific countries where there are no parameters related to trust and media use. Second, the index used in this study represents social vulnerability at the individual and national levels; therefore, it is not practical for guiding community-based actions to minimize vulnerability. Third, we used a single factor analysis model for each data set to develop the index, so the changes in SVI values across different time periods should be interpreted with caution.

### Conclusions

The SVI, constructed from a comprehensive list of socioeconomic and sociopsychological variables, can predict compliance with WHO advice on protective behaviors against COVID-19. Substantial gaps in social vulnerability to comply with the WHO advice existed at baseline and were exacerbated after the Omicron wave in African and Asia Pacific countries. The SVI developed in this study, particularly for African countries, could be used to identify vulnerable populations and monitor the progress of policy responses to minimize social barriers to adopting protective behaviors against COVID-19. This study can contribute to the design of prevention measures for future public health emergencies.

## Appendix

Multimedia Appendix 1 Supplementary tables and figures.

## Abbreviations

GDP: gross domestic product
HALE: health-adjusted life expectancy
SVI: social vulnerability index
WHO: World Health Organization

## Data Availability

The data sets generated and/or analyzed during the current study are available from the corresponding author on reasonable request.
