# Supplementary material for: Social Vulnerability and Compliance With World Health Organization Advice on Protective Behaviors Against COVID-19 in African and Asia Pacific Countries: Factor Analysis to Develop a Social Vulnerability Index
Source: JMIR Public Health Surveill. 2024 Aug 13;10:e54383. doi: 10.2196/54383 (PMC11350317; doi:10.2196/54383)
Supplement: Multimedia Appendix 1 [file publichealth_v10i1e54383_app1.docx]

**Table S1 Characteristics of indicators used for SVI construction and data sources.**

| **Indicators** | **AFCs** | **APCs** | **Source of data** |
| --- | --- | --- | --- |
| Age | 1) 18-24 years old  2) 25-34 years old  3) 35-44 years old  4) 45-54 years old  5) 55+ years | 1) 18-24 years old  2) 25-34 years old  3) 35-44 years old  4) 45-54 years old  5) 55+ years | AFCs  Nationally representative surveys carried out by the Vaccine Confidence Project in January 2022 (baseline) and August 2022 (follow-up).  APCs  Nationally representative surveys carried out by the Vaccine Confidence Project between June and August 2021 (baseline), and May and June 2022 (follow-up). |
| Gender | 1) Male  2) Female | 1) Male  2) Female |  |
| Education | 1) No formal education  2) Primary education  3) Secondary education  4) Vocational post-secondary education/Others  5) University/Tertiary education  6) Masters/PhD | 1) No formal education  2) Primary education  3) Secondary education  4) Vocational post-secondary education  5) University/Tertiary education  6) Masters/PhD |  |
| Employment status | 1) Unemployed (no income)  2) Retired, student, stay-at-home parent (no own income from work)  3) Working parttime, self-employed, jobs other than working fulltime (irregular income)  4) Working fulltime (regular income) | 1) Unemployed (no income)  2) Retired, student, stay-at-home parent (no own income from work)  3) Working parttime, self-employed, jobs other than working fulltime (irregular income)  4) Working fulltime (regular income) |  |
| Urban | 1) Urban  2) Rural | 2021 survey  NA  2022 survey  NA |  |
| Trust in government | 1) Not at all  2) Not much  3) Somewhat  4) A lot | NA |  |
| Trust in healthcare personnel | 1) Not at all  2) Not much  3) Somewhat  4) A lot | 2021 survey  NA  2022 survey  NA |  |
| COVID19 risk perception | A. Level of COVID19 threat pose to you?  1) Very high threat  2) Fairly high threat  3) Moderate threat  4) Fairly low threat  5) Very low threat  6) No threat  B. How concerned are you about getting COVID19?  1) Not at all  2) A little  3) Moderate  4) Very | How strongly do you agree or disagree that the threat from COVID-19 is exaggerated?  1) Strongly Agree  2) Agree  3) Disagree  4) Strongly disagree |  |
| Media use and communication | How often do you use the following (television, radio, print media, word of mouth in the local community, social media, the internet)?  1) Every day  2) At least once a week  3) At least once a month  4) Less than once a month  5) Never | Not available |  |
| Population using internet (%) | Not included because internet use variable obtained from the surveys. | Latest update | https://ourworldindata.org/internet |
| GDP per capita | 2021 for baseline and follow up | 2020 for baseline and 2021 for follow up | <https://data.worldbank.org/indicator/NY.GDP.PCAP.CD> |
| Nursing and midwifery personnel per 10,000 population (because nurses are at the front line in both hospitals and communities and play an important role in tackling COVID19(61) | Latest update | Latest update | https://www.who.int/data/gho/data/indicators/indicator-details/GHO/nursing-and-midwifery-personnel-(per-10-000-population) |
| Healthy life expectancy at birth | Latest update | Latest update | https://www.who.int/data/gho/data/indicators/indicator-details/GHO/gho-ghe-hale-healthy-life-expectancy-at-birth |
| Population with basic handwashing facilities at home (%) | 2020* | Not included due to a large proportion of missing data | https://www.who.int/data/gho/data/indicators/indicator-details/GHO/population-with-basic-handwashing-facilities-at-home-(-) |
| Population density (%) | 2022 for baseline and follow up | 2021 for baseline and 2022 for follow up | https://ourworldindata.org/grapher/population-density |

Note: * Data for South Sudan were not available, so population access to improved sanitation indicator was used instead (data available from https://www.unicef.org/southsudan/media/9321/file/WASH%20Briefing%20Note_2021%20Q4.pdf.

**Figure S1 Factor loadings**

1. **AFCs baseline**

**Factor loadings (pattern matrix) and unique variances**


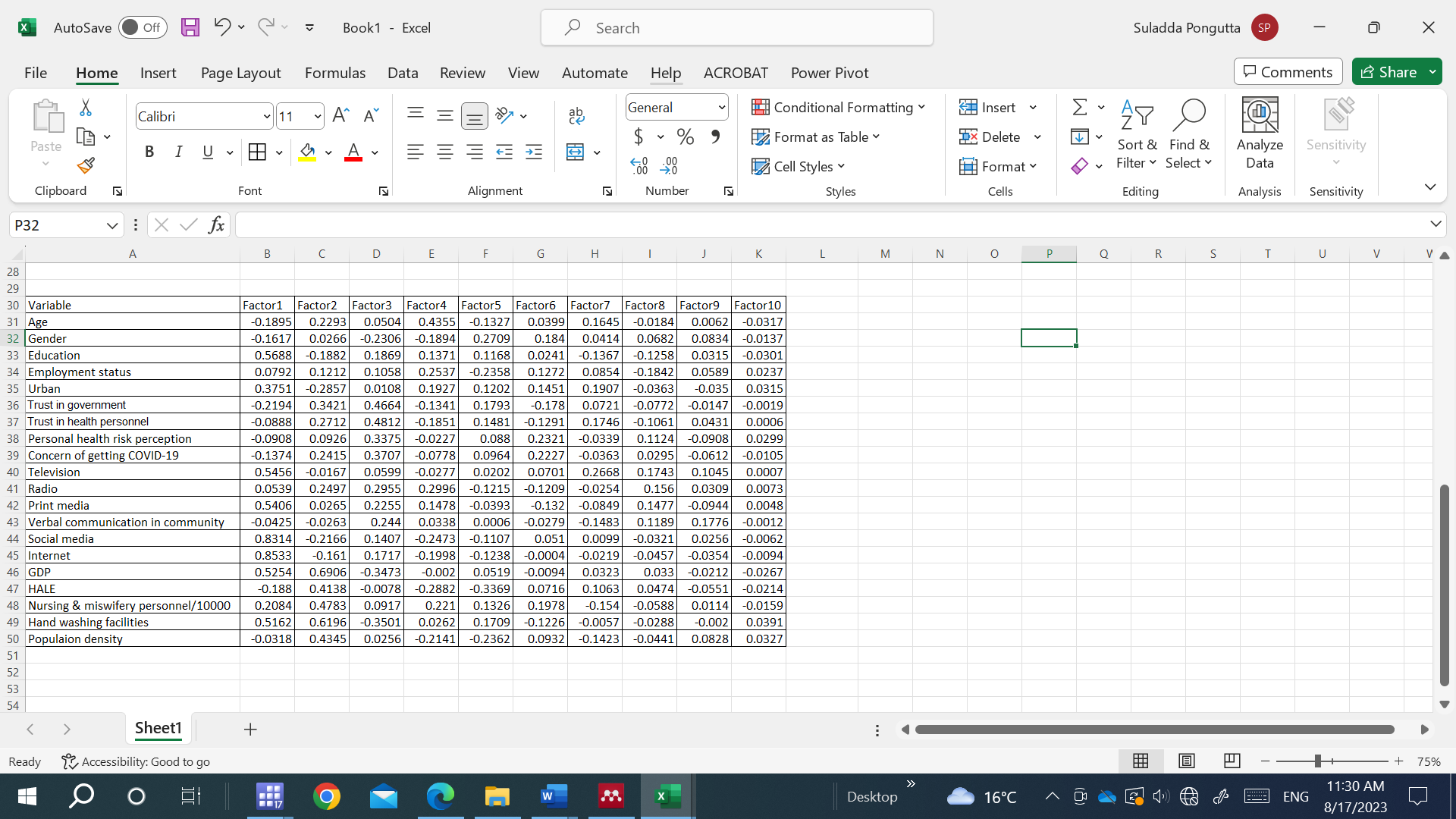


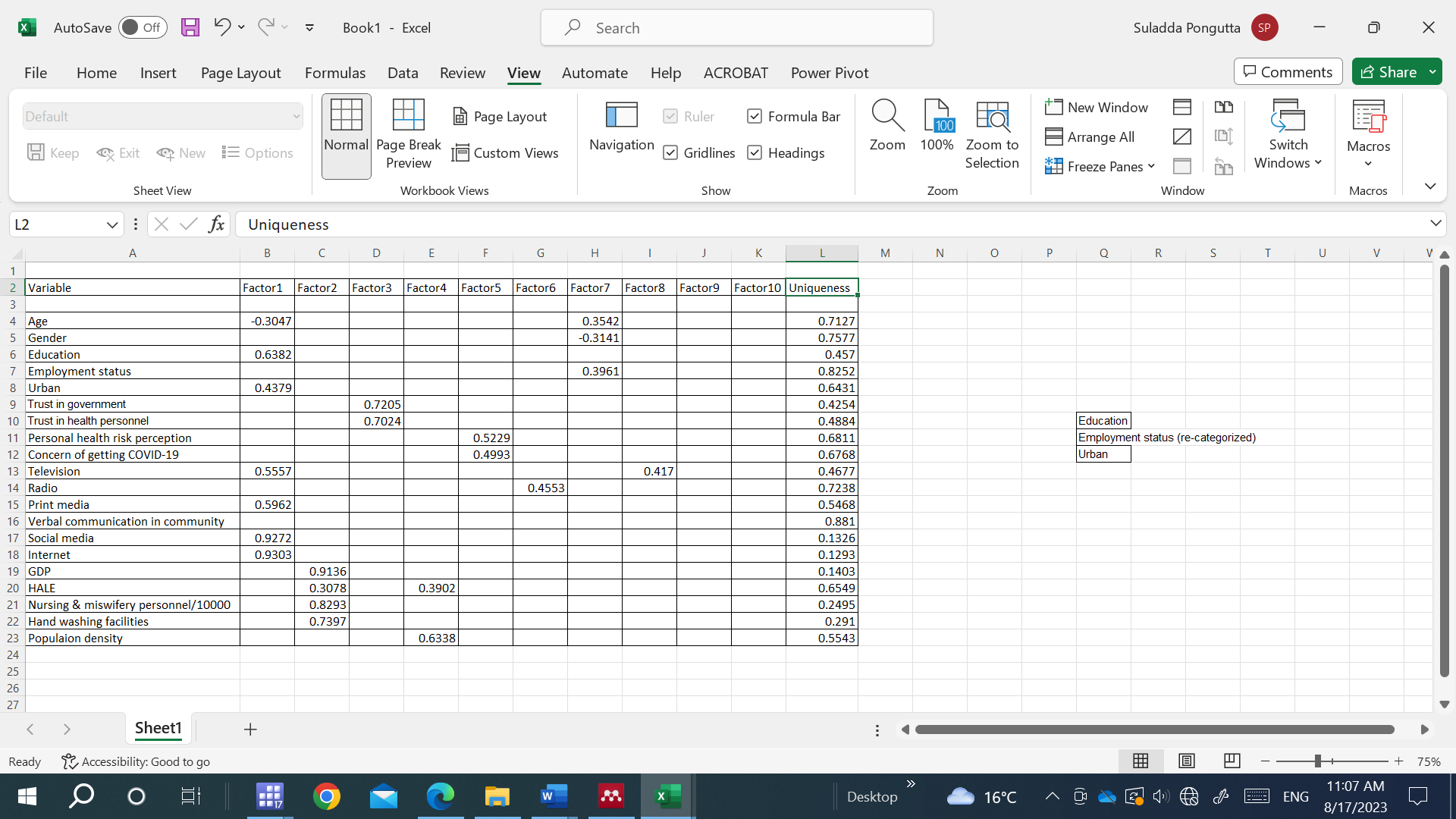


Overall Kaiser-Meyer-Olkin (KMO) = 0.72

1. **AFCs follow-up**

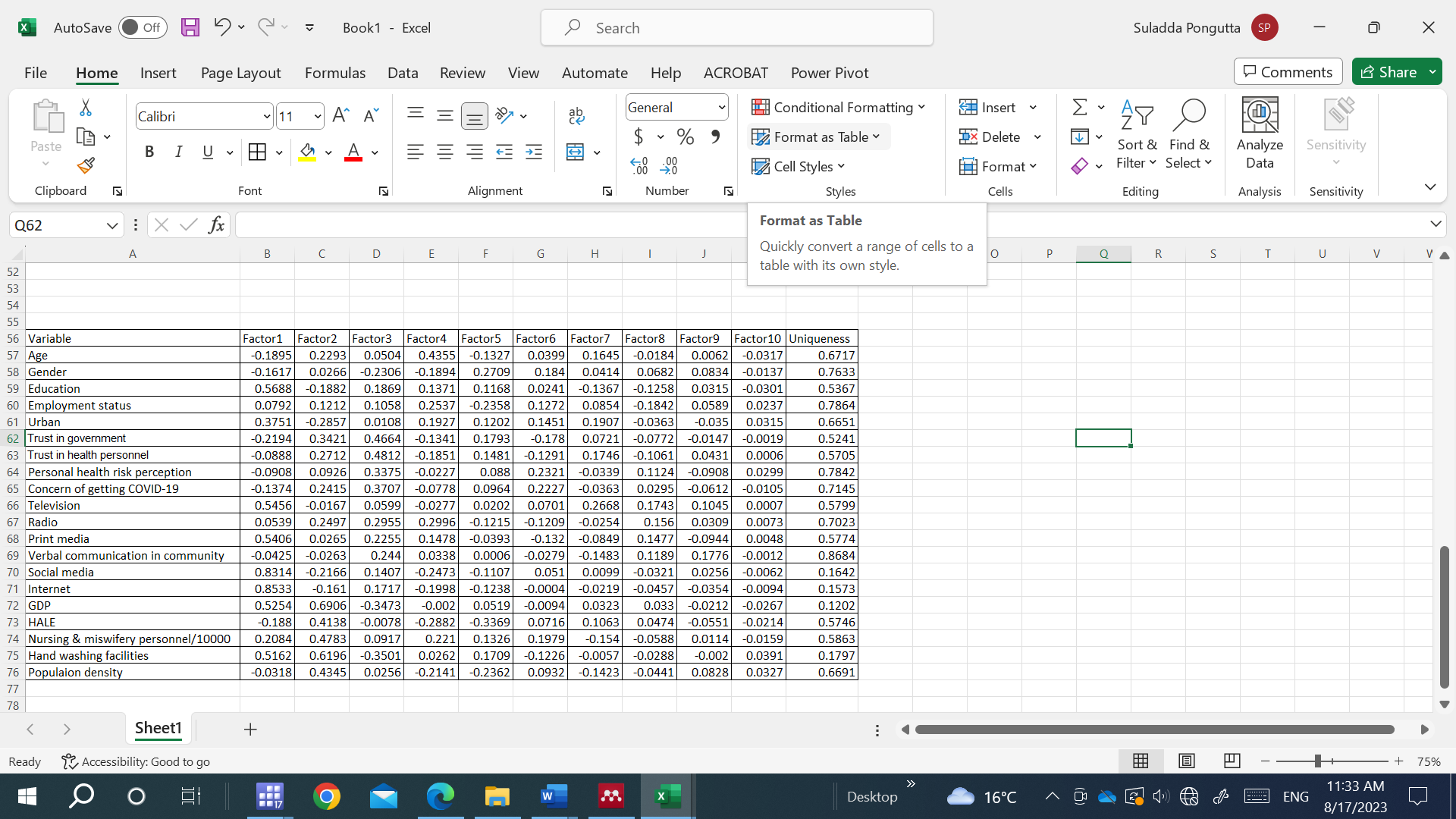

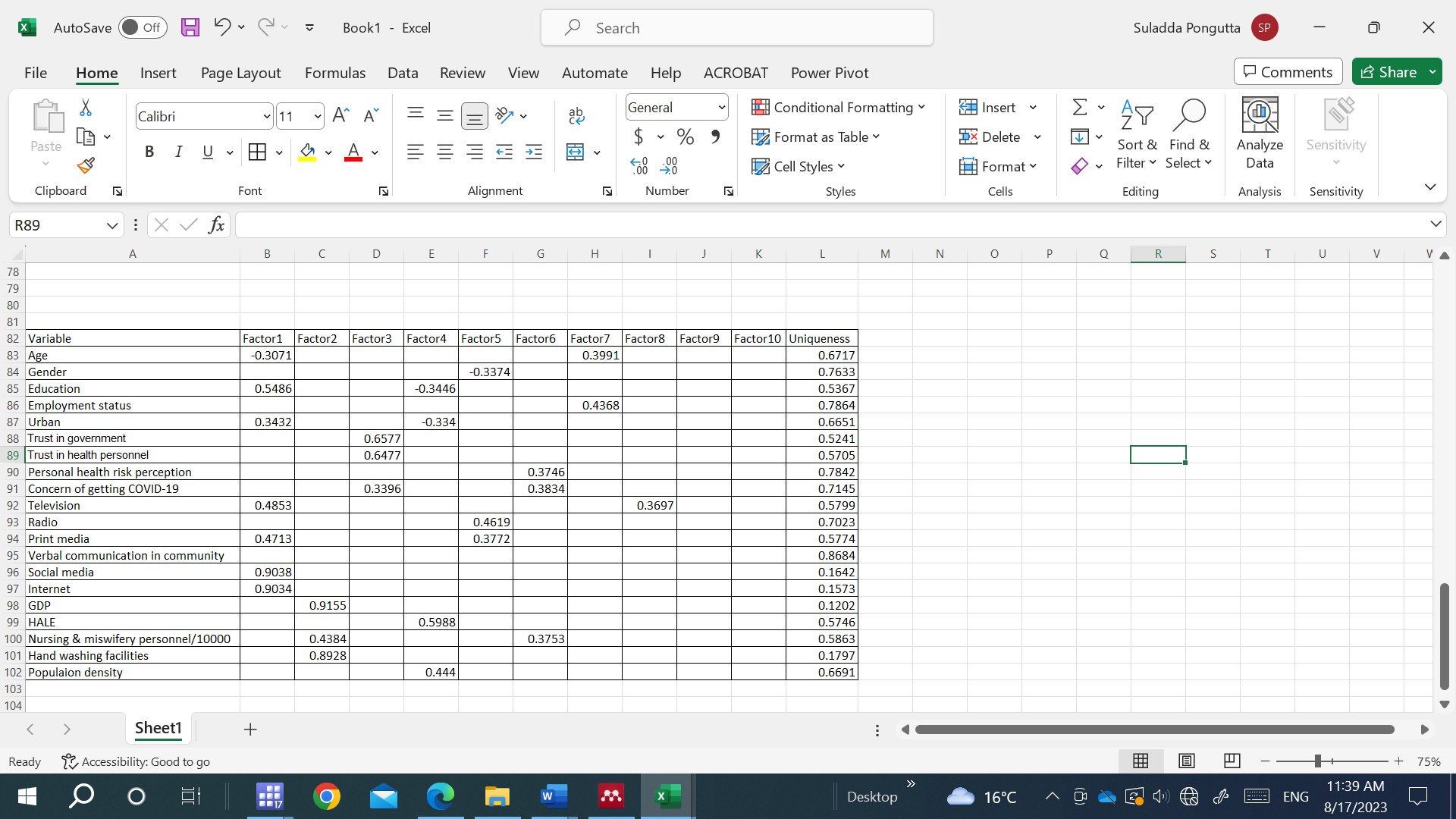


Overall Kaiser-Meyer-Olkin (KMO) = 0.70

1. **APCs baseline**

Factor loadings (pattern matrix) and unique variances


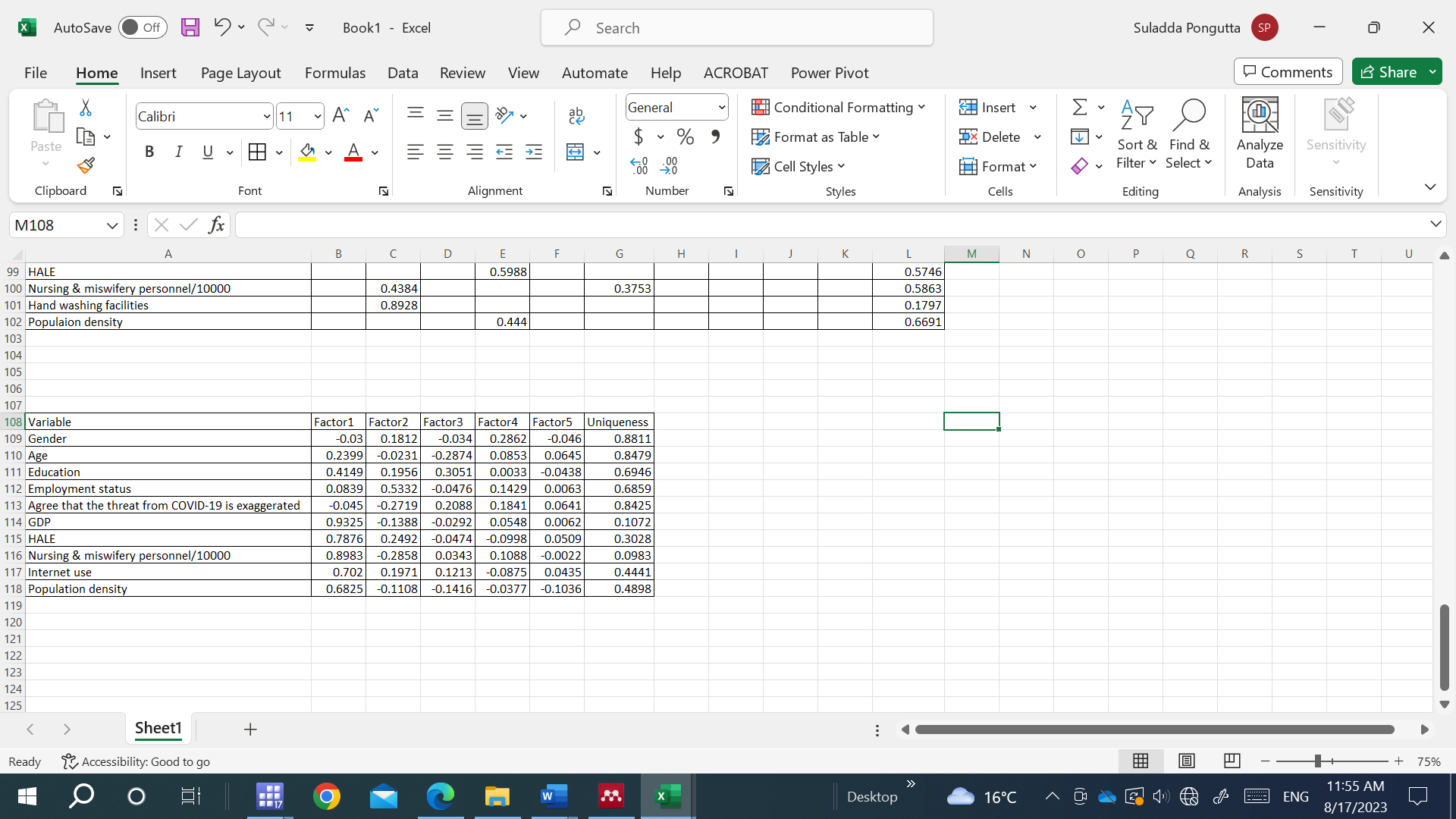

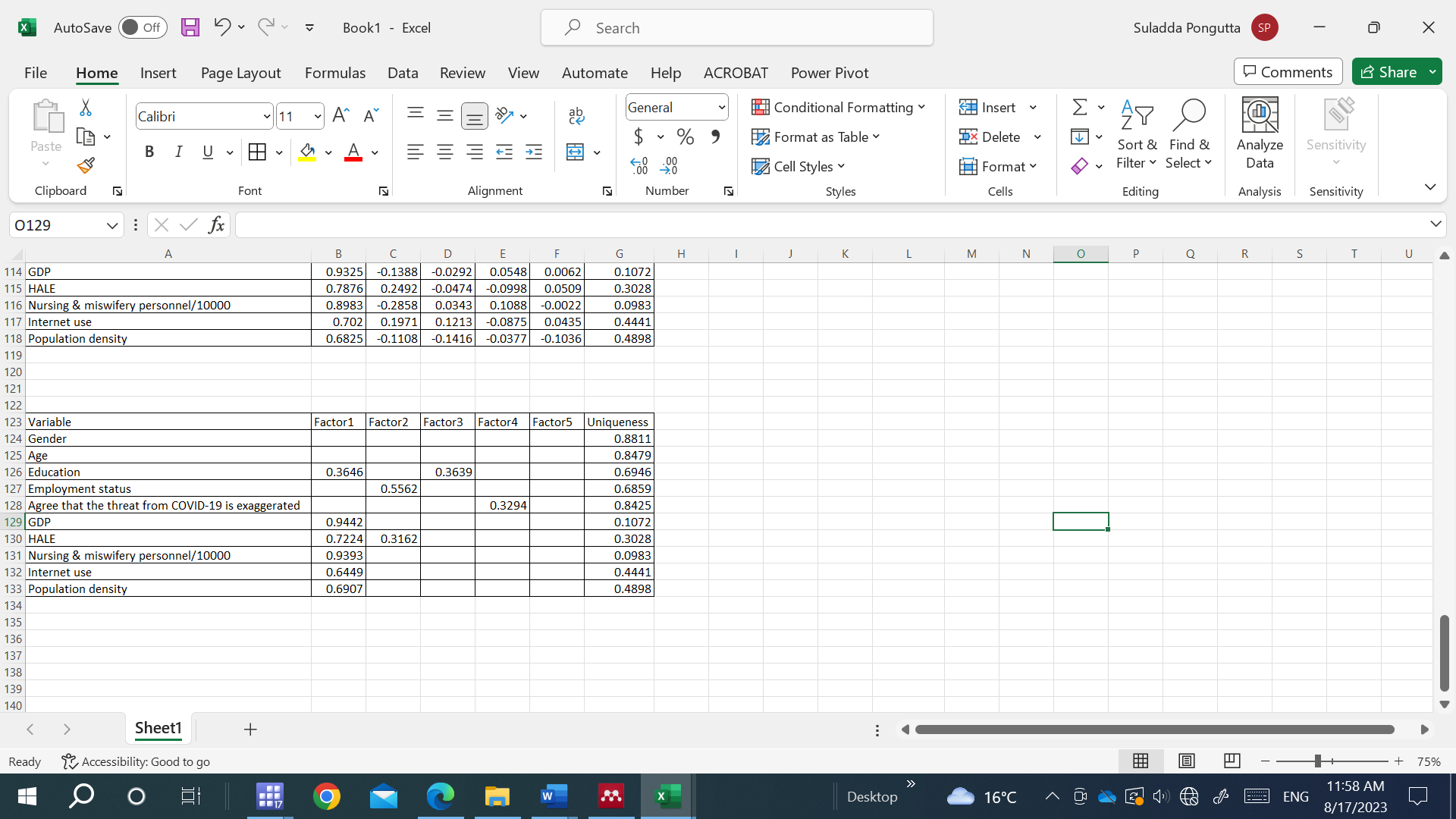


Overall Kaiser-Meyer-Olkin (KMO) = 0.81

1. **APCs follow-up**

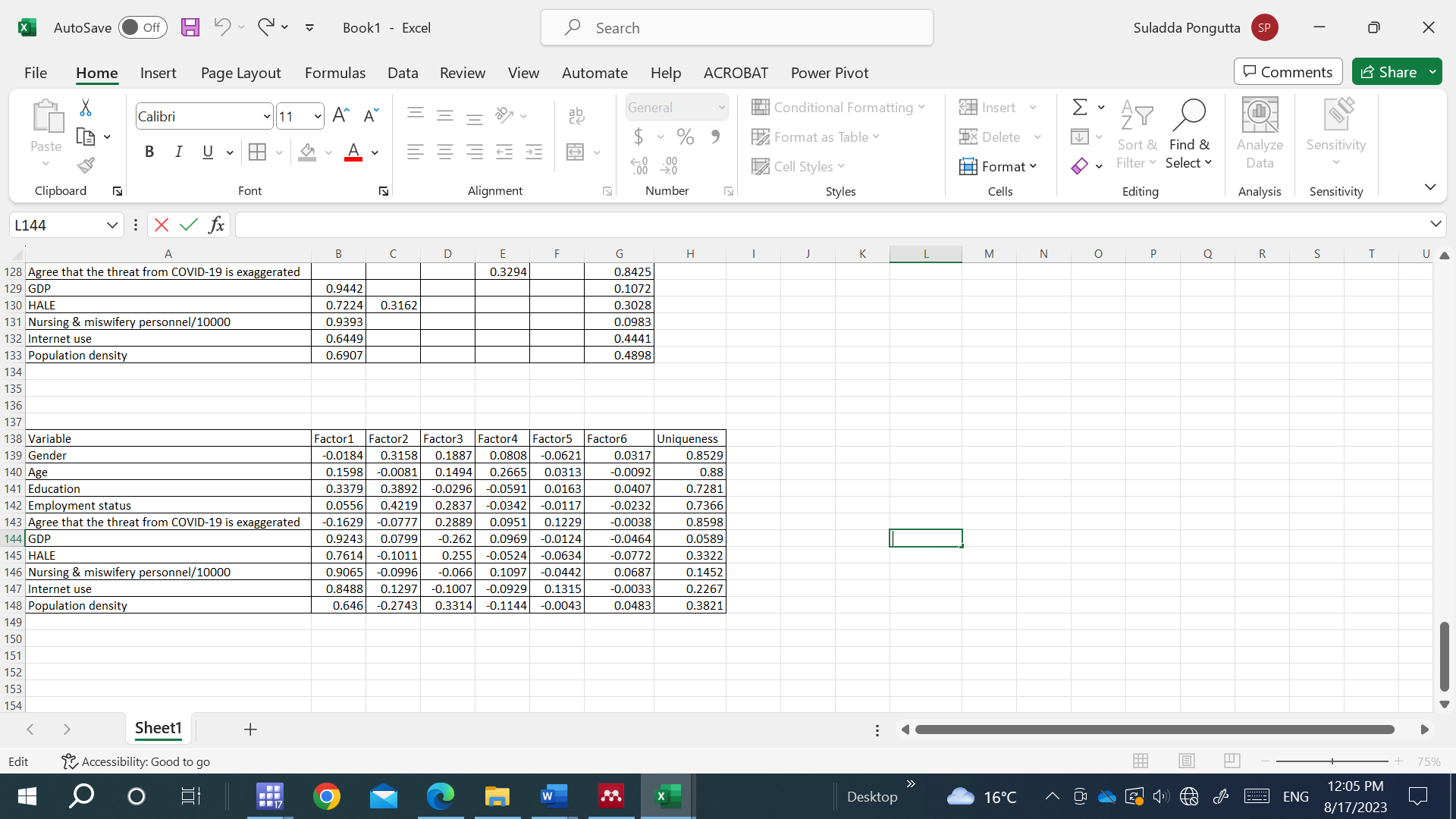

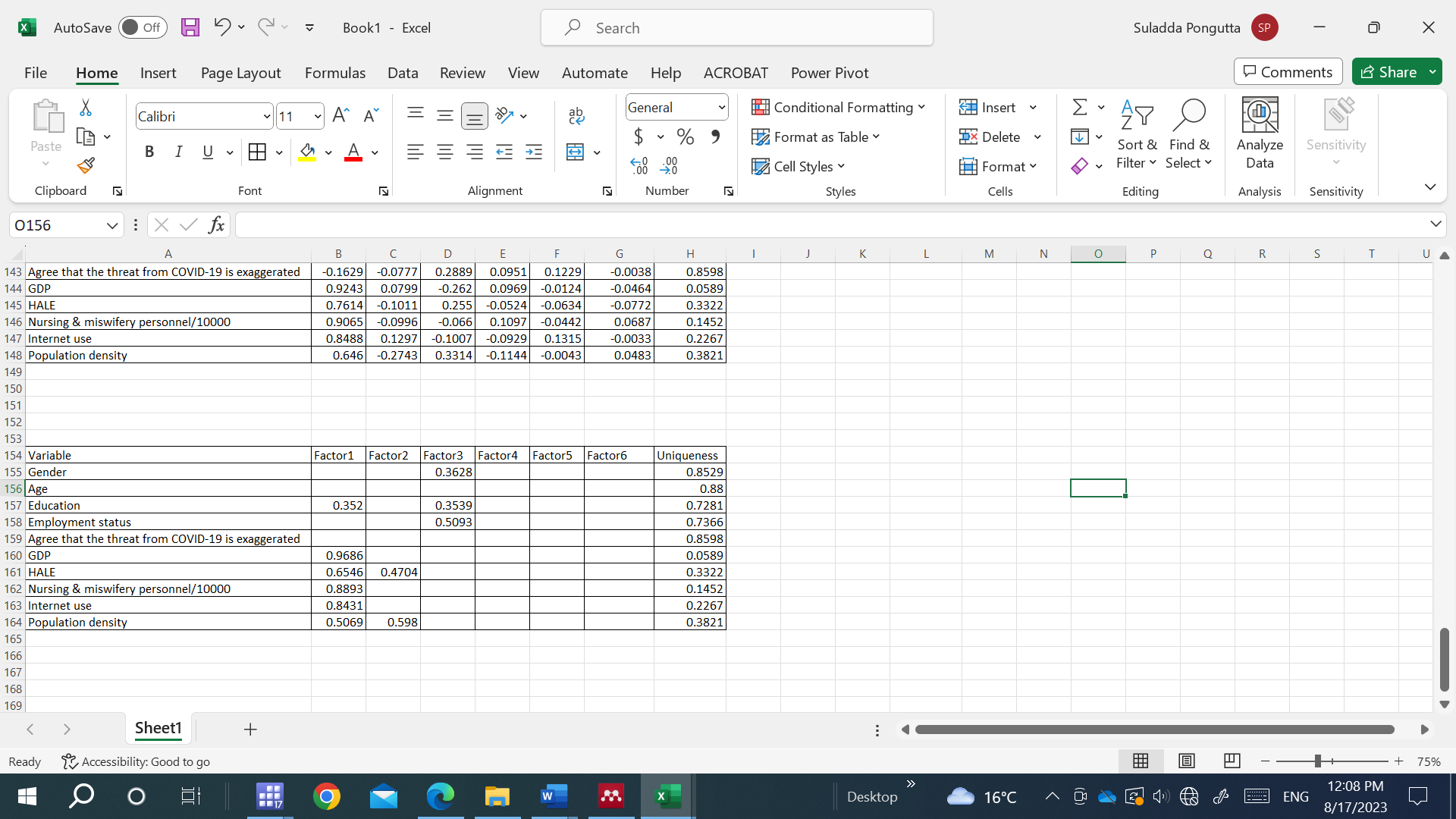


Overall Kaiser-Meyer-Olkin (KMO) = 0.72
